# Reagent-free total protein quantification of intact extracellular vesicles by attenuated total reflection Fourier transform infrared (ATR-FTIR) spectroscopy

**DOI:** 10.1007/s00216-020-02711-8

**Published:** 2020-05-29

**Authors:** Veronika Szentirmai, András Wacha, Csaba Németh, Diána Kitka, Anita Rácz, Károly Héberger, Judith Mihály, Zoltán Varga

**Affiliations:** 1 Biological Nanochemistry Research Group, Institute of Materials and Environmental Chemistry, Research Centre for Natural Sciences, Magyar tudósok körútja 2, Budapest, 1117 Hungary; 2 Plasma Chemistry Research Group, Institute of Materials and Environmental Chemistry, Research Centre for Natural Sciences, Magyar tudósok körútja 2, Budapest, 1117 Hungary

**Keywords:** Extracellular vesicle (EV), Infrared spectroscopy, Protein quantification, ATR-FTIR, Chemometrics

## Abstract

Extracellular vesicles (EVs) are lipid bilayer–bounded particles that are actively synthesized and released by cells. The main components of EVs are lipids, proteins, and nucleic acids and their composition is characteristic to their type and origin, and it reveals the physiological and pathological conditions of the parent cells. The concentration and protein composition of EVs closely relate to their functions; therefore, total protein determination can assist in EV-based diagnostics and disease prognosis. Here, we present a simple, reagent-free method based on attenuated total reflection Fourier transform infrared (ATR-FTIR) spectroscopy to quantify the protein content of EV samples without any further sample preparation. After calibration with bovine serum albumin, the protein concentration of red blood cell–derived EVs (REVs) were investigated by ATR-FTIR spectroscopy. The integrated area of the amide I band was calculated from the IR spectra of REVs, which was proportional to the protein quantity in the sample‚ regardless of its secondary structure. A spike test and a dilution test were performed to determine the ability to use ATR-FTIR spectroscopy for protein quantification in EV samples, which resulted in linearity with *R*^2^ values as high as 0.992 over the concentration range of 0.08 to 1 mg/mL. Additionally, multivariate calibration with the partial least squares (PLS) regression method was carried out on the bovine serum albumin and EV spectra. *R*^2^ values were 0.94 for the calibration and 0.91 for the validation set. The results indicate that ATR-FTIR measurements provide a reliable method for reagent-free protein quantification of EVs.

**Electronic supplementary material:**

The online version of this article (10.1007/s00216-020-02711-8) contains supplementary material, which is available to authorized users.

**Figure:**
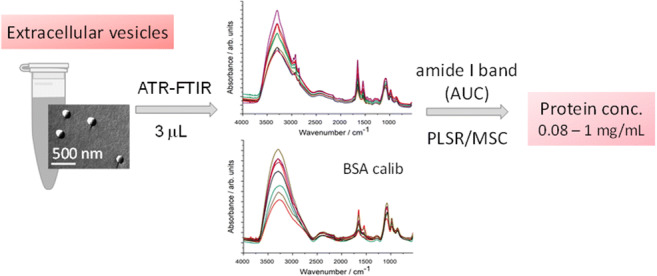
Graphical abstract

## Introduction

Extracellular vesicles (EVs) are heterogeneous lipid bilayer–bounded particles created and secreted by cells [1, 2]. Their common feature is their size range from 30 nm up to several micrometers [3] and their biological functions: communication and biomaterial transport between cells through the extracellular space [4]. Not surprisingly, EVs can be found in every biofluid, e.g., in lymph, blood, urine, saliva, breast milk, and spinal fluid, and they can give information about the physiological and pathological conditions of the parent cells. This makes them potential new biomarkers enabling a so-called liquid biopsy [5, 6]. Moreover, based on the role of EVs in biomaterial transport between cells, their applicability as drug delivery vehicles is also under investigation [7].

Despite the exponentially growing research in the EV field, reliable and reproducible characterization methods are still lacking. EV quantification is still a major issue: determination of particle number and/or total protein amount are the most commonly used approaches for the assessment of the amount of EVs [8]. Most of the protein determination assays are dye-binding assays (bicinchoninic acid—BCA or Bradford, for example) based on different color changes of a dye in response to various concentrations of proteins [9]. Colorimetric assays are widely used; however, different assays may provide different concentration values for the same sample. As an example, on appropriate dilutions of gravimetrically prepared 10 mg/mL solutions of bovine serum albumin (BSA), Biuret, Lowry and Bradford assays provided results of 9.7, 8.4 and 21.1 mg/mL protein concentrations, respectively [10]. Moreover, in the case of EVs, results may depend on whether detergent is used to disrupt the vesicles to expose the whole protein content. Infrared spectroscopy is a label-free, highly reproducible, and sensitive analytical method feasible to complement or even replace standard protein quantification procedures [11–13]. Especially the attenuated total reflection (ATR) technique has a great potential regarding protein determination in serum and whole blood enabling potential clinical applications [14–16]. IR spectroscopy of EVs, however, was promoted only in the last few years. Our research group was a pioneer in the application of ATR-FTIR spectroscopy as a reagent-free, non-invasive and simple measurement mode to study extracellular vesicles [17]. Based on selected amide and C–H stretching band intensity ratios, we proposed a “spectroscopic protein-to-lipid ratio” which provides a useful index for EV characterization [8, 17–19].

The purpose of the present study is to assess the feasibility of ATR-FTIR spectroscopy as a fast and simple method for EV’s protein quantification based on particular IR bands and range.

Red blood cell (RBC)–derived EVs (REVs) were used as model EVs. RBCs produce a large amount of EVs through shedding microvesicles to get rid of specific harmful agents that tend to accumulate during their lifespan. REVs are produced both in vivo and ex vivo: in the latter case, REVs can be isolated easily by centrifugation from the supernatant of stored RBC concentrates, which results in a homogeneous population of EVs from a single cell origin with uniform composition and size distribution.

## Materials and methods

### Extracellular vesicle isolation and characterization

Whole blood (3 × 6 mL) was collected from healthy volunteers into blood collection vacuum tubes containing EDTA anticoagulant (Greiner Bio-One, VACUETTE® TUBE 6 mL K3EDTA). The use of human blood samples was approved by the Scientific and Research Ethics Committee of the Hungarian Medical Research Council (ETT TUKEB 6449-2/2015). During the entire investigation, we followed the guidelines and regulations of the Helsinki Declaration in 1975. Cellular components were sedimented by centrifugation (Nüve NF800R, swing-out rotor, 2500×*g*, 15 min, 4 °C) and after removing the plasma (supernatant) and the white blood cell containing buffy coat, the RBCs were washed at least 3 times with 0.9% NaCl physiological saline solution (2500×*g* for 10 min at 4 °C). 3.5 mL of washed RBCs were diluted to 10 mL with phosphate-buffered saline (PBS, pH = 7.4, Sigma-Aldrich, Hungary) and stored at 4 °C for 7 days.

REVs were isolated by the removal of cells from RBC suspension by two consecutive sedimentations (1500×*g* for 10 min and 2850×*g* for 30 min, respectively). The supernatant was then aliquoted into Eppendorf tubes (2 mL) and pelleted at 16000×*g* for 30 min (Eppendorf 5415R, F45-24-11 rotor). The final pellets were suspended in 200 μL PBS and purified with size exclusion chromatography (SEC) using a 3.5 mL gravity column filled with Sepharose CL-2B gel (GE Healthcare, Sweden). One hundred microliters of REV sample was pipetted onto the column followed by the addition of 900 μL PBS. The second 1 mL fraction containing the purified REVs were collected and kept at 4 °C until further investigations (max. 48 h) [17, 20, 21].

The morphology of REVs in their native hydrated structure was visualized by freeze-fracture combined electron microscopy (FF-TEM). REV samples were mixed with glycerol (at 3:1 sample:glycerol volume ratio) to avoid freezing artifacts. Approximately 2 μL of sample was pipetted onto a golden sample holder and suddenly frozen in freon at − 196 °C, then stored in liquid nitrogen. Fracturing was performed at − 100 °C in a Balzers freeze-fracture device (Balzers BAF 400D, Balzers AG, Liechtenstein). Replicas from the fractured surfaces were made by carbon-platinum shadowing, then washed with surfactant solution and distilled water. The replicas were placed on a 200-mesh copper grid and examined in a MORGAGNI 268D (FEI, The Netherlands) transmission electron microscope.

Counting and sizing of REVs were performed by microfluidic resistive pulse sensing (MRPS), a method based on the Coulter principle, and implemented on a microfluidic chip. MRPS measurements were performed with an nCS1 instrument (Spectradyne LLC, USA) using factory calibrated TS-400 cartridges with a measurement range from 65 to 400 nm. The samples were diluted 20-fold with bovine serum albumin (BSA, Sigma-Aldrich, Hungary) solution at 1 mg/mL in PBS buffer (pH = 7.4, Sigma-Aldrich, Hungary), filtered through an Amicon Ultra 0.5 mL MWCO100 kDa membrane filter (Merck Millipore, Germany) according to the manufacturer’s instructions.

### Protein concentration determination by colorimetric methods (Bradford and bicinchoninic acid assays)

Total protein content of samples was determined using the Bio-Rad Protein Assay, based on Bradford’s method that involves the binding of Coomassie Brilliant Blue G-250 dye to proteins. Bovine serum albumin (BioRad Laboratories, USA) was used as standard (2 mg/mL). Ten microliters of the sample and 250 μL dye (1× Bradford reagent) was added to a 96-well plate and the absorbance was measured at 595 nm. Bicinchoninic acid assay (Pierce BCA Protein Assay Kit, Thermo Scientific, USA) was performed in a similar way: 25 μL of the samples were combined with BCA working reagent (200 μL) and incubated at 37 °C for 30 min in a 96-well plate before reading at 562 nm. BCA working reagent was prepared by mixing 50:1 volume ratio of BCA stock solution and 4% CuSO_4_ × 5H_2_O prior to the beginning of the assay. All UV-Vis measurements were done at room temperature using a BioTek Synergy 2 plate reader.

### Attenuated total reflection Fourier transform infrared spectra collection and analysis

ATR-FTIR spectra were collected using a Varian 2000 FTIR Scimitar Series spectrometer (Varian Inc., USA) equipped with a liquid nitrogen–cooled mercury-cadmium-telluride (MCT) detector and with a “Golden Gate” single reflection diamond ATR accessory (Specac Ltd., UK). Sixty-four co-added scans, covering a wavenumber range of 4000–550 cm^−1^, were collected using a spectral resolution of 2 cm^−1^ by the ResolutionPro 4.0 software. Due to the strong water absorbance of the IR light, air-dried samples are generally preferred over liquid samples [11, 16]. Three microliters of sample was mounted on the diamond ATR crystal and a thin dry film was obtained by slow evaporation of the solvent under ambient conditions. The measurements were performed at room temperature, immediately after drying the sample (within approximately 10 min, controlled by real-time monitoring of water bands to achieve a steady-state intensity). After each data acquisition, ATR correction was performed.

Raw IR spectra should be pre-processed by baseline correction and normalization. The following spectral manipulation steps were applied by an in-house software:

- The baseline correction was determined at 2575, 1905, 1380, 1215, and 605 cm^−1^ from 64 spectra during the preliminary measurements of BSA calibration and EV samples as the relevant minima in the spectra being within ± 5 cm^−1^ of these values. Rubber-band baseline correction [22] was performed. All spectra were normalized with respect to the intensity of the highest absorption band of phosphate buffer at 1070 ± 10 cm^−1^.
- The phosphate buffer spectrum was subtracted using the “de-wiggle” algorithm by iteratively minimizing the first derivative of the subtraction result [23, 24] for the 1850–1350 cm^−1^ wavenumber region of the spectrum. This step was adequate to remove efficiently the overlapping OH bending of buffer from the amide I region.
- The area under the curve (AUC) for the amide I was calculated by integration of 1700–1600 cm^−1^ spectra region.

### Univariate statistics

Protein concentrations are expressed as mean ± SD. Statistical analysis was carried out using one-way ANOVA, followed by Tukey’s multiple comparison by using the GraphPadPrism 7.04 software (GraphPad Software Inc.). The cut-off for statistical significance was set at *p* < 0.05 (*p < 0.05).

### Multivariate statistics

The multivariate calibration model based on bovine serum albumin samples has been established by partial least squares regression, PLSR [25, 26]. There is no need to explain the algorithm of PLSR thoroughly here; a detailed tutorial can be found in ref. [25]. PLSR and similar latent variable methods used in pharmaceutics were integrated with “advanced characterization techniques such as vibrational spectroscopy” [26].

The baseline-corrected ATR-FTIR spectra of the samples were applied for the PLSR model building and the protein concentration of the REV samples was predicted with the model. In this case, multiplicative scatter correction (MSC) was used as extra spectral pre-processing for the IR spectra of the samples. Threefold cross-validation with randomized sampling was applied as the validation method for the modeling. It means that each third of the samples is predicted as the test set once and only once, while the other two-thirds are used as the calibration set. *R*^2^ values and root mean squared error for calibration and validation values were calculated as performance metrics for the model.

## Results and discussion

### Red blood cell–derived extracellular vesicles

**Fig. 1:**
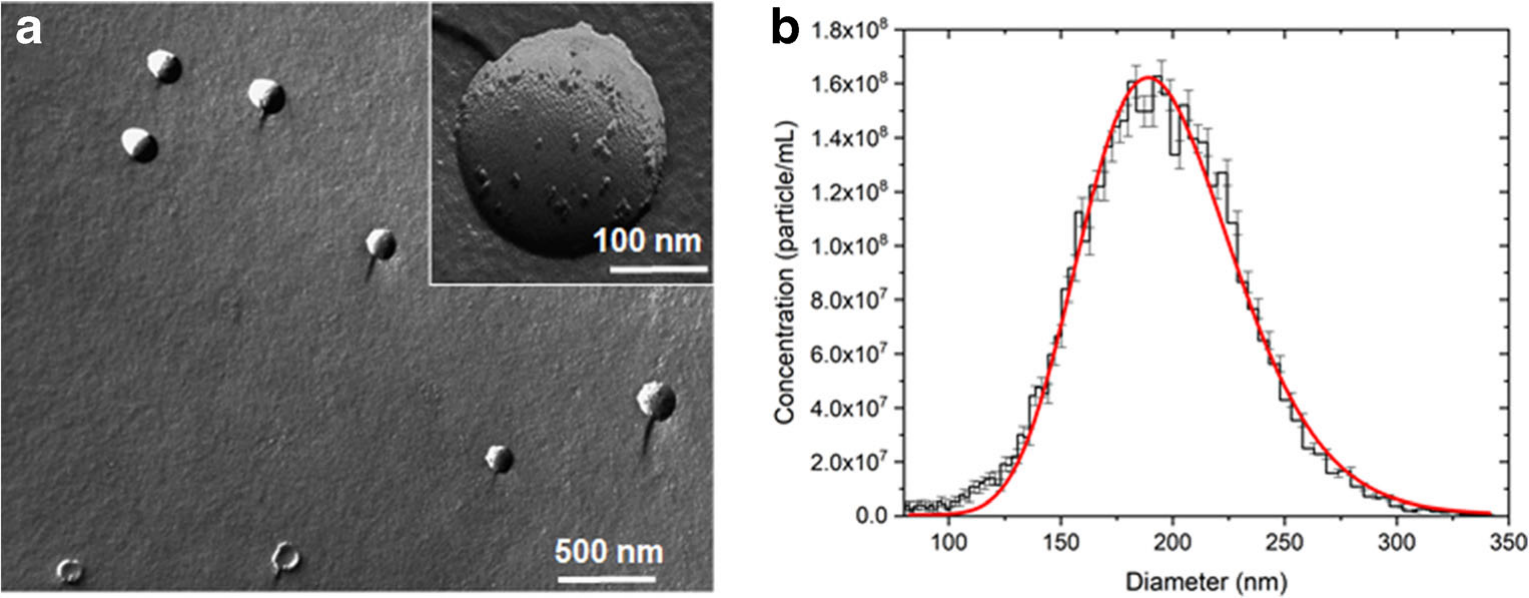
Red blood cell–derived extracellular vesicles (REVs). **a** Freeze-fracture combined transmission electron microscopy (FF-TEM) picture (in inset an enlarged image of a single REV). **b** Size distribution and concentration of REVs measured by microfluidic resistive pulse sensing (MRPS)

The presence and quality of REVs in the samples was investigated by FF-TEM and MRPS measurements. FF-TEM, by prompt freezing of the sample, enables the direct visualization of REVs’ morphology in their native structure (Fig. 1a). Spherical vesicles of 150–250 nm diameter with speckled surface can be observed in the TEM picture.

The size distribution and concentration of REVs were measured by MRPS [20]. Measured data points were fitted by a log-normal distribution (Fig. 1b) with a mean diameter of 195 ± 0.6 nm (adj. *R*^2^ = 0.979; *μ* = 198 ± 0.6 nm and *σ* = 35.7 ± 0.6 nm). The measured concentration is (1.44 ± 0.01) × 10^10^ particle/mL over a size range from 65 to 400 nm.

### IR spectra of EV samples

**Fig. 2:**
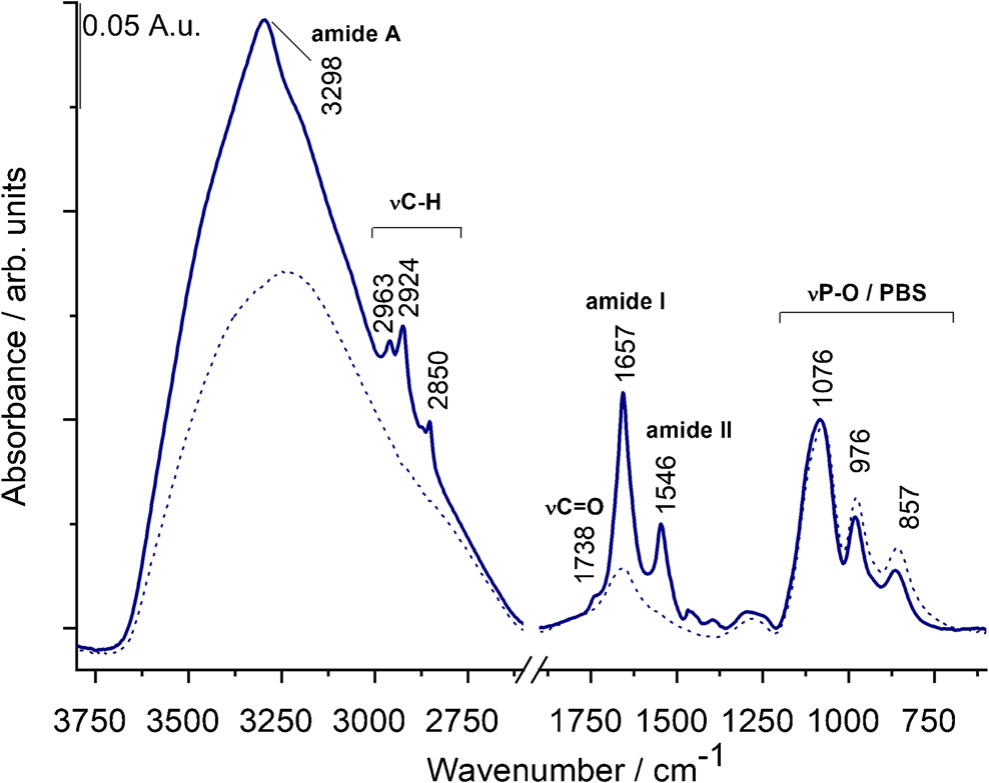
Typical spectrum of a REV sample with characteristic bands of proteins and lipids. The spectrum of PBS buffer is also presented with dotted line

Difficulties in the analysis of EV samples by IR spectroscopy may arise from the usually low EV concentration of the samples and the presence of contaminants such as lipoproteins, protein aggregates, etc. with similar IR features as EVs. However, the protein-to-lipid ratio might be a quality control of EV purity [8, 18]. Based on the ratio of the amide and C–H stretching band intensities, the “spectroscopic protein-to-lipid ratio” can serve as a useful index for EV characterization [17]. Figure 2 shows a typical IR spectrum of REV samples. The use of the dry film technique with ATR configuration produces high-quality spectra of EVs with clearly defined spectral features [17, 27]. Characteristic protein bands of amide A, amide I, and amide II can be found at 3298, 1657, and 1546 cm^−1^, respectively. Vibrations corresponding to lipid components can also be witnessed as antisymmetric and symmetric methylene stretching of acyl chains at 2924 cm^−1^ and 2850 cm^−1^, respectively, and the C=O stretching at 1738 cm^−1^ of the glycerol esters. In the low-frequency region, however, the phosphate bands of PBS buffer are dominating the spectrum due to the low EV concentration. It should be noted that the buffer has also a contribution to the amide I band (Fig. 2, dotted line spectrum) so any quantification based upon amide I band intensity might be corrected by proper buffer subtraction. The “spectroscopic protein-to-lipid” ratio calculated as the ratio of integrated areas of the amide I band and that of the C–H stretching (from 3040 to 2800 cm^−1^ wavenumber region) resulted in a value of 1.2 ± 0.1 for REV samples, which is in line with our previous results [20].

### Protein standard plot

**Fig. 3:**
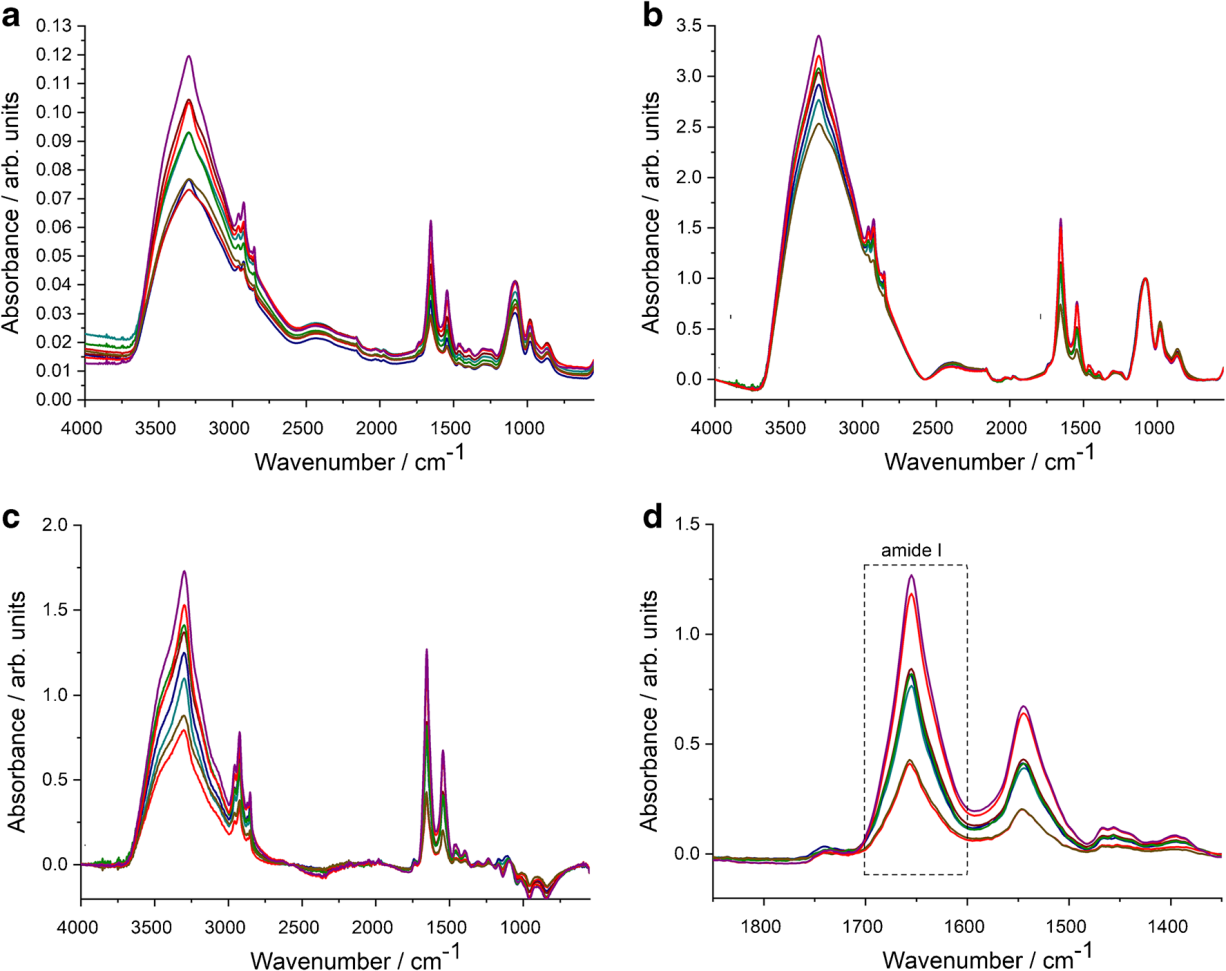
Summary of spectral pre-processing steps demonstrated by spectra of REV samples. **a** Raw spectra after ATR correction. **b** Spectra after baseline correction and normalization. **c** Spectra after buffer subtraction. **d** Zoomed spectra for the calculation of area under the curve (AUC) values of the amide I band by integration in 1700–1600 cm^−1^ wavenumber region

**Fig. 4:**
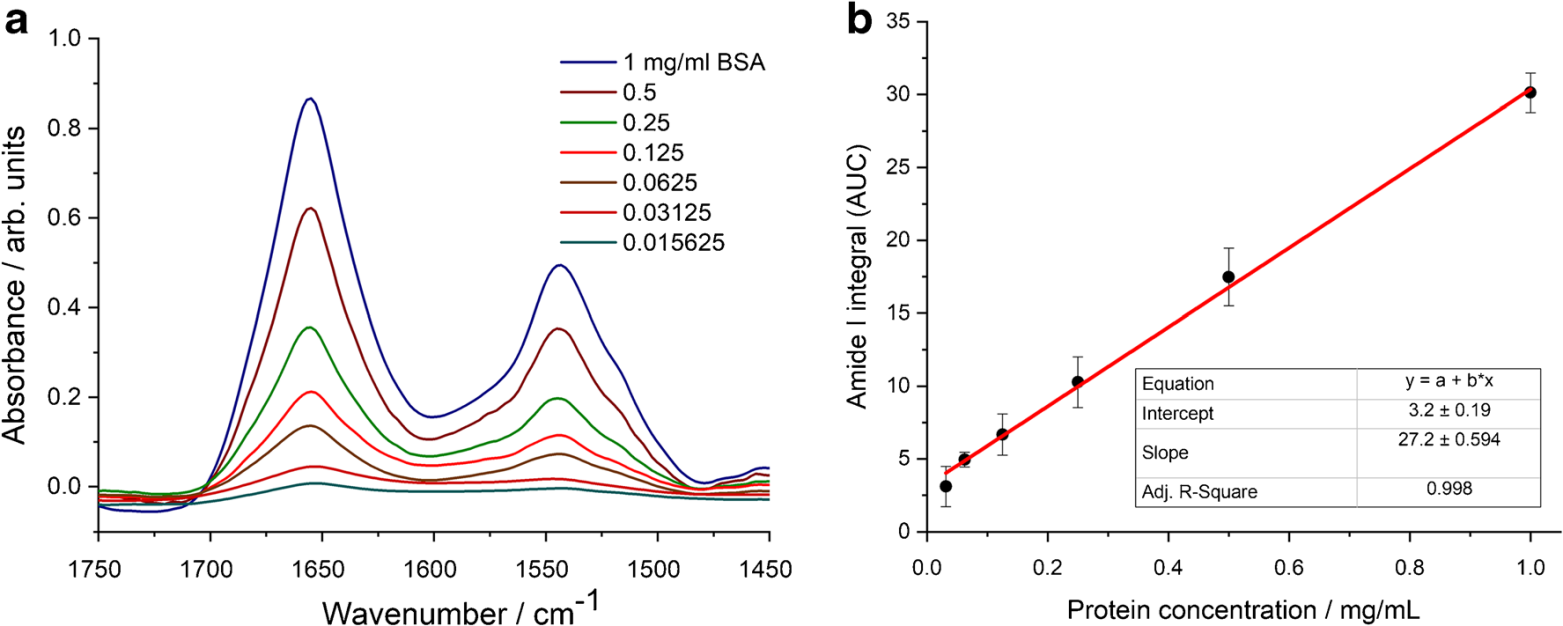
ATR spectra of BSA standards in the amide I and amide II region (**a**) and the generated calibration curve used for protein quantification (amide I AUC plot versus concentration) (**b**). Error bars denote standard deviation (SD) obtained from three parallel measurement including drying on the ATR surface, data acquisition and spectral manipulation

As protein amide I and amide II are usually well-defined IR bands of EV samples, protein quantification based upon a simple univariate (Lambert-Beer) analysis is feasible. The integrated area of amide I is related to the protein concentration through a calibration curve. We have to note that recent papers dealing with ATR-FTIR spectroscopy–based protein quantification relay on multivariate analysis [16]. Despite the general trend of solving analytical calibration problems by multivariate statistics and chemometric methods [28–30], the peculiar feature of intact EV samples, the novelty of the IR spectroscopy in the EV field, and the effort towards a simple and standardisable EV quantification method rationalize the use of a simple univariate calibration method [31, 32]. All IR spectra of the reference protein and REV samples were treated with the pre-processing and analysis protocol described in the “Materials and methods” section and illustrated in Fig. 3. Bovine serum albumin (BSA) was used as protein standard for the generation of the calibration plot. In line with the expected EV concentration, 1 mg/mL BSA dissolved in PBS buffer was used as stock solution and a dilution series (by sample bisections) was applied to create a calibration curve for the protein concentration determination (Fig. 4a and b).

Good linearity was obtained in the protein range between 0.031 and 1 mg/mL (covering almost two orders of magnitude) with adjusted *R*^2^ value of 0.998 (Fig. 4b). The last dilution point (0.016 mg/mL) was not considered for the calibration curve. Indeed, the bottom spectrum (Fig. 4a) does not show distinctive amide I and amide II bands which would be needed for IR-based protein quantification [33]. The linear relationship between concentration and the amide I AUC was validated by the experimental *F* value corresponding to the ratio of residual variance to squared pure error [30]. Calculated for a heteroscedastic case, we obtained an *F* value of 0.7886 which proved to be significantly lower than the critical *F* value of 2.6896 [34], reconfirming the linearity of our calibration curve (Electronic Supplementary Material (ESM) Fig. S1). Sensitivity of the method was verified by calculating the limit of detection (LOD) and the limit of quantification (LOQ) involving a certain risk of false positives (false detects, *α*-errors) and false negatives (false non-detects, *β*-errors) [30, 35]. Working at 95% confidence level, the probability of *α*-errors and *β*-errors, *α* and *β*, respectively, are usually reasonable small values (*α* = *β* = 0.05) and the univariate LOD and LOQ can be expressed as

$$ LOD=\frac{3.3 Sy/x}{slope}\sqrt{1+{h}_0+\frac{1}{n}}\kern0.5em LOQ=\frac{10 Sy/x}{slope}\sqrt{1+{h}_0+\frac{1}{n}} $$

[30, 35], where *Sy/x* is the residual standard deviation, *n* is the number of calibration samples, and *h*_0_ is the leverage for the blank sample, calculated from the mean calibration concentration. LOD was found to be 0.03 mg/mL, while LOQ 0.08 mg/mL. These values are somewhat higher compared with colorimetric-based protein quantification methods (under ideal circumstances the detection limit of Bradford and BCA assays is at 0.02 mg/mL [36]), however, it is acceptable for ATR-FTIR-based protein quantification [12, 15].

### Adaptation and validation for REV samples

Proteomics of REV samples identified more than 200 different proteins for ex vivo excreted EVs from stored red blood cells [37]. However, since the FTIR-based protein quantification is related to the number of amide bonds (on mass basis) a single protein could serve as an adequate reference for any other proteins [13]. Furthermore, noting that the band 3 protein, one of the key protein of RBCs and REVs [37], has a molecular weight of 93,000 g/mol and contains 833 amino acid residues [38], we can calculate an approximate peptide bond concentration as 0.00895 per volume (L). This value coincides well with that of BSA being 0.00912 per volume (L). As further proof, Strug and co-workers [13] compared three different types of proteins (BSA, protein A, and rabbit IgG) and they obtained a similar slope for amide I intensity versus protein concentration, independent of protein sequence content.

**Fig. 5:**
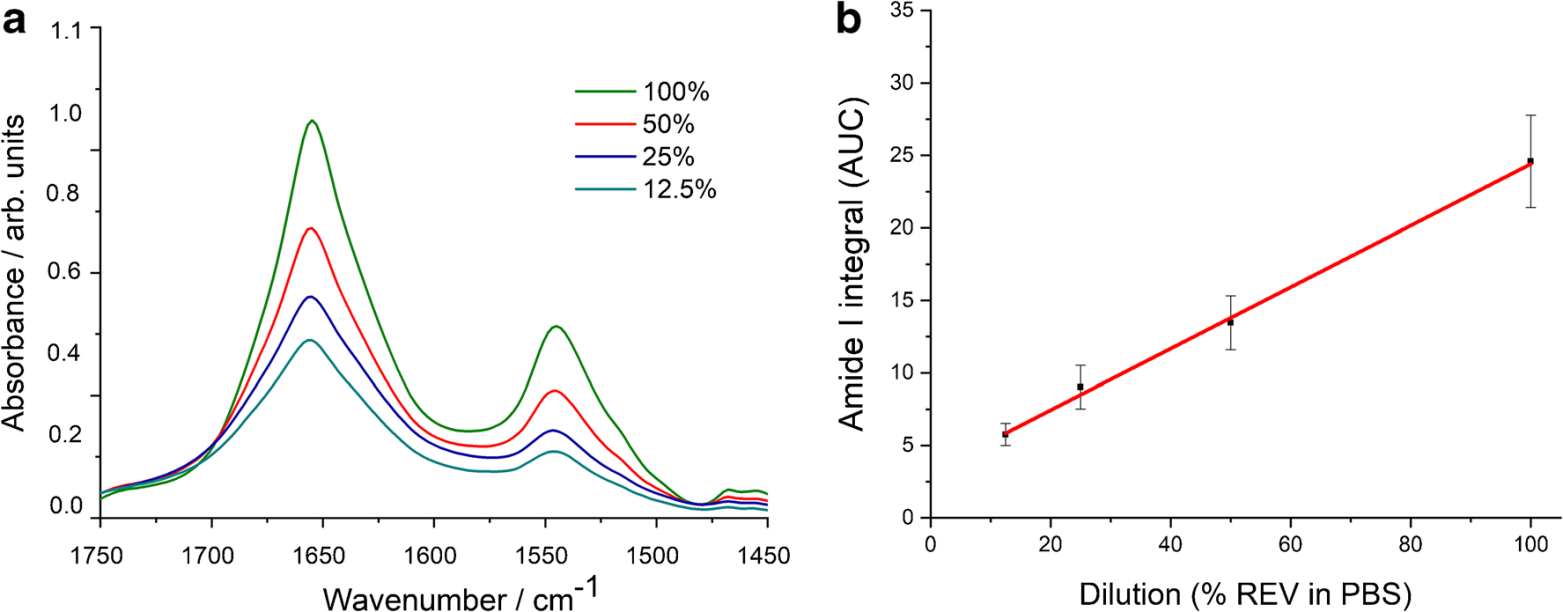
**a** ATR spectra of REV sample from the dilution test. Amide I and amide II band intensities decrease with dilution (presented as % REV in PBS). **b** amide I AUC plot vs REV dilution (presented as % REV in PBS). Error bars denote standard deviation (SD) obtained from three parallel measurement including drying on the ATR surface, data acquisition, and spectral manipulation

In order to test the adaptability of the method to EVs, the linearity of amide I band area (AUC) with REV concentration was verified. Figure 5a shows the amide I and amide II wavenumber region of the pre-processed IR spectra of REV samples after different dilutions.

The dilution curve (Fig. 5b) calculated from the integrated area of the amide I bands shows a very good linear dependence upon dilution (adj. *R*^2^ value of 0.992). Again, performing the *F* test [34] the obtained *F* value of 0.2924 compared with the critical *F* value of 4.4590 corroborates the linearity (ESM Fig. S2). Upon dilution, the estimated uncertainty of the data points decreases and the standard deviation yields SD values under 1% proving the precision of measurements. For the undiluted REV sample, we obtained a higher standard deviation with an SD value of 23.27%, but which is still acceptable. The reason for the data precision failure is, that around 1070 cm^−1^, dominated by the phosphate vibration of the PBS buffer, EV components might have also minor contributions (e.g., the stretching vibrations of the phosphodiester groups of phospholipids; the C–O–C stretching vibrations of phospholipids, triglycerides, and cholesterol esters) [17, 39]. The iteration-based spectral subtraction protocol aims to overcome this adverse effect; however, the PBS subtraction is still the bottleneck of the process. However, this effect is marginal form a practical point of view due to the usually low concentration of EV samples.

The proposed EV protein quantification method is further validated by a spike-and-recovery test. Due to their relatively large surface-to-volume ratio, EV samples might be prone to adsorb proteins that are present in the biological matrix during EV preparation. For example, blood plasma–derived EVs might have substantial amounts of albumin as external protein cargo [40, 41]. To assess possible interference of REVs with albumin, a known amount of REV sample was spiked with low, medium, and high concentration of BSA standard solution (0.0625, 0.25, and 1 mg/mL BSA,respectively). To avoid undesired dilution, the REV sample was concentrated by centrifugation (16,000×*g* for 10 min) beforehand to obtain a higher initial concentration (1.07 ± 0.13 mg/mL) and was spiked with BSA solution in a volume ratio of REV:BSA = 8:2. Spike recovery calculated as (total concentration detected − concentration original)/concentration spiked × 100% [42] resulted in a mean recovery of 100 ± 5.2% for 0.25 and 0.0625 mg/mL BSA spikes. The mean recovery was lower (92 ± 5.4%) for the high amount of BSA spike; in this case, the total protein concentration is over the dynamic range of the calibration and saturation of absorption intensity of protein amide I band may occur. Within the dynamic range of the calibration, however, a good recovery rate was obtained providing that the interference between an external protein cargo and the proposed EV total protein quantification is negligible.

### Multivariate modeling with PLSR

**Fig. 6:**
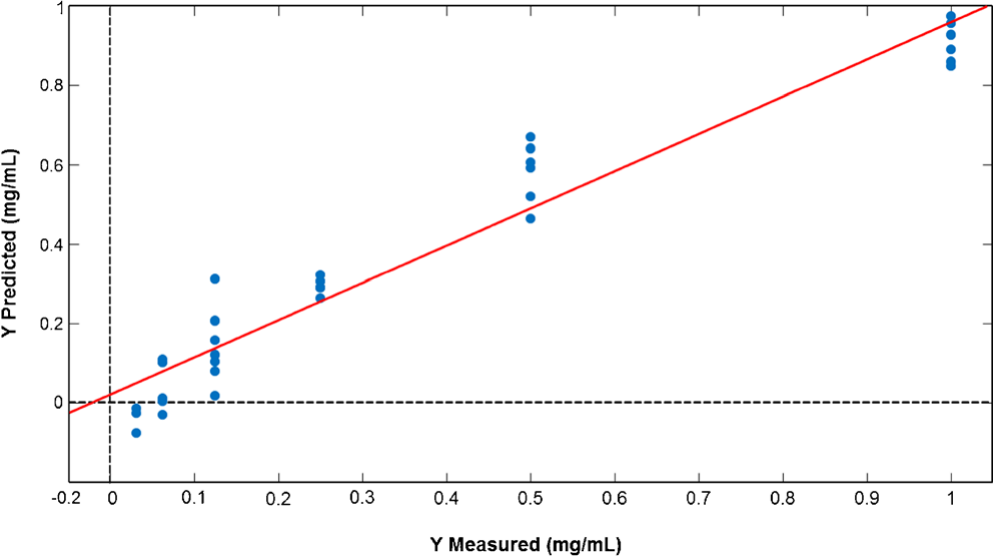
Result of measured vs predicted protein concentrations (mg/mL) in the case of cross-validation. The curve for the cross-validation set is displayed as a red line

For the sake of thoroughness, we have also carried out multivariate modeling on the spectral dataset with the PLS regression method. Thirty-three BSA samples were included in the data matrix and the previously mentioned amide 1 and amide II regions of the spectra (1750–1450 cm^−1^) were selected for modeling. Additionally, the spectra were scaled with the MSC method. Threefold cross-validation with randomized sampling was applied in the validation phase of the modeling and four PLS components were included in the final model. The number of PLS components was determined by the local minimum of the root mean squared error of cross-validation (RMSECV) curve. The *R*^2^ values of the calibration (*R*^*2*^_*C*_) and validation set (*R*^*2*^_*CV*_) were 0.94 and 0.91, respectively. The error of the final model was given by the root mean squared error of calibration (RMSEC) and cross-validation (RMSECV): 0.08 mg/mL for the calibration and 0.10 mg/mL for the validation, respectively. The measured and predicted protein concentration for the validation set is reported in Fig. 6. A permutation test was also applied for the validation of the model. The result showed that the model significantly differs from the use of random numbers, based on a 100-iteration protocol.

The final model shows satisfactory performance, thus we predicted the total protein concentrations of the REV samples from three independent blood sources (donors) and independent isolations (REV1, REV2, and REV3). In the case of the REV1 sample, a dilution series was also prepared (Fig. 5).

**Fig. 7:**
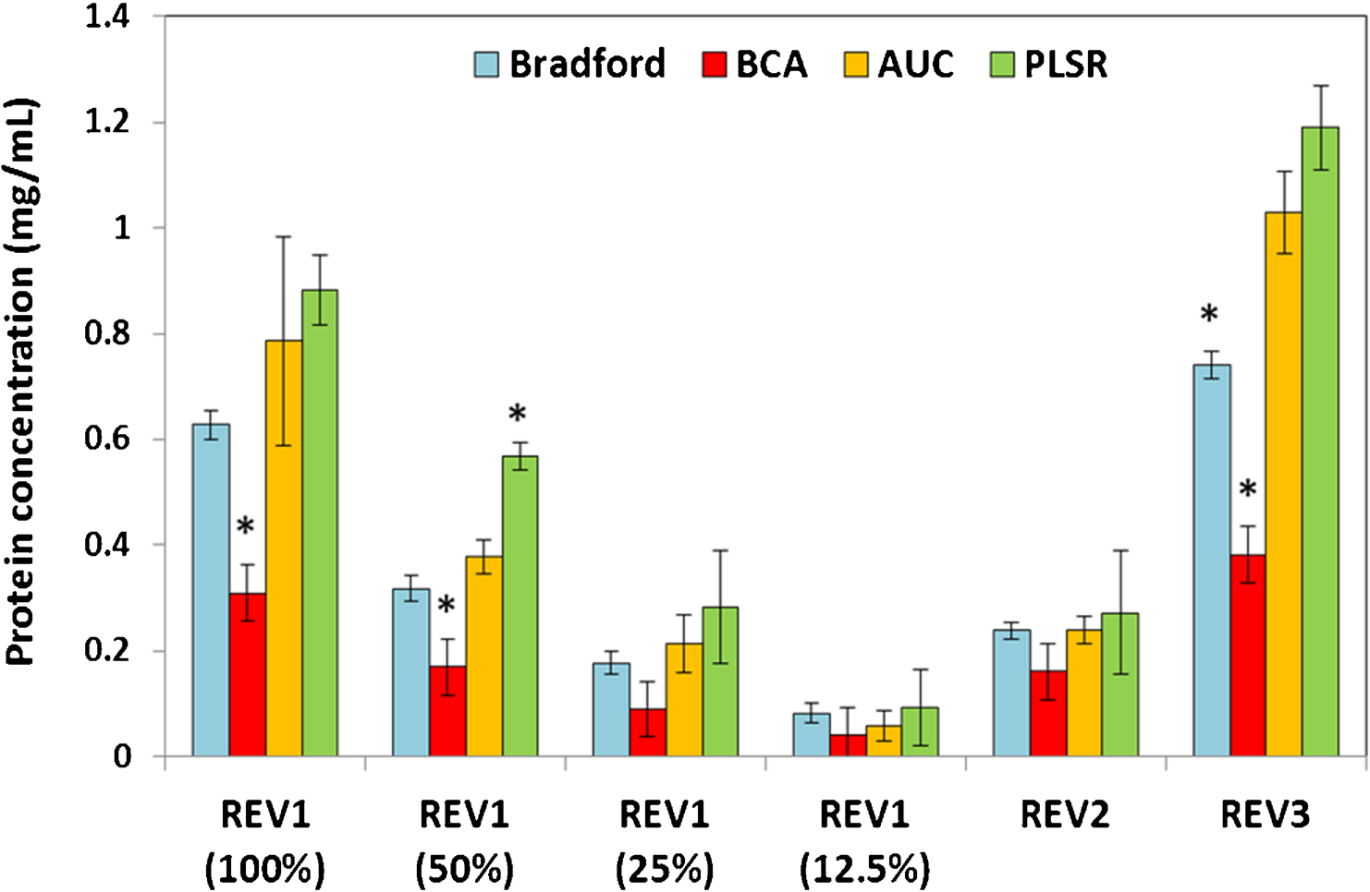
Comparison of total protein concentrations (presented as mean ± SD) of REV samples obtained from different sources (different donors). Assays providing significantly different concentration values (based on Tukey’s test) are marked with stars for each sample. AUC means the amide I band area–based determination, while PLSR is the PLS regression–based determination

Finally, the total protein content of REV samples was determined by the ATR-FTIR methods in the AUC-based and the multivariate (PLSR) workflows, as well. The results were compared with the outcomes of the Bradford and BCA assays (Fig. 7). The linearity of FTIR-based determinations was clearly shown by REV1 dilutions. By performing Tukey’s post hoc statistical test, we obtained only one significant difference between the results of the Bradford protein assay and the ATR-FTIR methods at a confidence interval of 95%. The two colorimetric assays, however, resulted in significantly different protein concentration values, especially in the higher concentration region (~ 50% less protein concentration measured by BCA). One possible explanation for the phenomenon should be the interference of bicinchoninic acid with phospholipids [43] and/or with reducing side chains present in REVs’ proteins.

Another issue is that due to the peculiar structure of EVs (stabile spherical vesicles) internal cargo proteins might be inaccessible to colorimetric dyes. Indeed, comparing the values obtained by the different methods, the highest protein concentration was measured by ATR-FTIR-based quantifications. Since IR spectroscopy is a label-free technique, it is not perturbed by the effectivity of dye-protein interaction. In the ATR-FTIR mode, the IR beam is directed through an internal reflection element (ATR crystal); an evanescent wave extends beyond the surface of ATR crystal and penetrates the sample. Since the penetration depth of this evanescence wave typically ranges from 1 to 2 μm within the 1800–900-cm^−1^ region [33], the whole protein content of REV samples (vesicles with average diameter of 200 nm) will be measured. A possible bias of the ATR-FTIR technique is related to the potential preferential adsorption of proteins onto the ATR crystal. REV sample disassembled by absolute ethanol was used to inspect colloidal aspects. The amide I areas calculated from the ATR-FTIR spectra of intact and lysed REV samples were similar (within LOQ range) even though the strong alteration in amide I band shape due to protein denaturation in the latter sample (ESM Fig. S3).

## Conclusion and outlook

IR spectroscopy, the ATR-FTIR technique is widely used for the analysis of biofluids including quantification of biomolecules of interest. In this study, we demonstrated that the IR spectroscopy–based protein quantification can be successfully adapted to the routine analysis of extracellular vesicles. The new method presents a reagent-free alternative to traditional colorimetric protein determination assays and requires no special sample preparation to investigate EVs.

The proposed spectrum analysis protocol based on the amide I band area, which implies the careful subtraction of the buffer spectrum, provides excellent linearity in the range of 0.08 to 1 mg/mL. Furthermore, multivariate modeling on raw spectral data set by Partial Least Squares regression (PLSR) combined with multiplicative scatter correction (MSC) also consolidates that protein quantification of extracellular vesicles can be conducted using this approach.

In our experiments, protein concentrations resulting from the IR spectroscopy–based method in majority are similar to that obtained using the Bradford assay. The bicinchoninic acid (BCA) assay, however, provided lower concentration values. Since no “gold standard” for total protein concentration of intact EVs exists, at this moment, we cannot draw further conclusion. However, due to its simplicity and minimal sample manipulation requirement, the protein concentration determination by ATR-FTIR spectroscopy might gain an important role in EV research. Compact ATR-FTIR instruments are already available and the proposed spectral analysis protocols for EV characterization can be readily automated. Thus, the ATR-FTIR-based protein quantification has great potential in clinical applications aiding EV-based diagnostics.

## Electronic supplementary material

ESM 1 (PDF 212 kb).
