# Supplementary material for: Reagent-free total protein quantification of intact extracellular vesicles by attenuated total reflection Fourier transform infrared (ATR-FTIR) spectroscopy
Source: Anal Bioanal Chem. 2020 May 29;412(19):4619–28. doi: 10.1007/s00216-020-02711-8 (PMC7329771; doi:10.1007/s00216-020-02711-8)
Supplement: Supplementary file 1 — (PDF 212 kb). [file 216_2020_2711_MOESM1_ESM.pdf]

Analytical and Bioanalytical Chemistry

Electronic Supplementary Material

Reagent-free total protein quantification of intact extracellular vesicles by attenuated total reflection Fourier transform infrared (ATR-FTIR) spectroscopy

Veronika Szentirmai, András Wacha, Csaba Németh, Diána Kitka, Anita Rácz,
Károly Héberger, Judith Mihály, Zoltán Varga

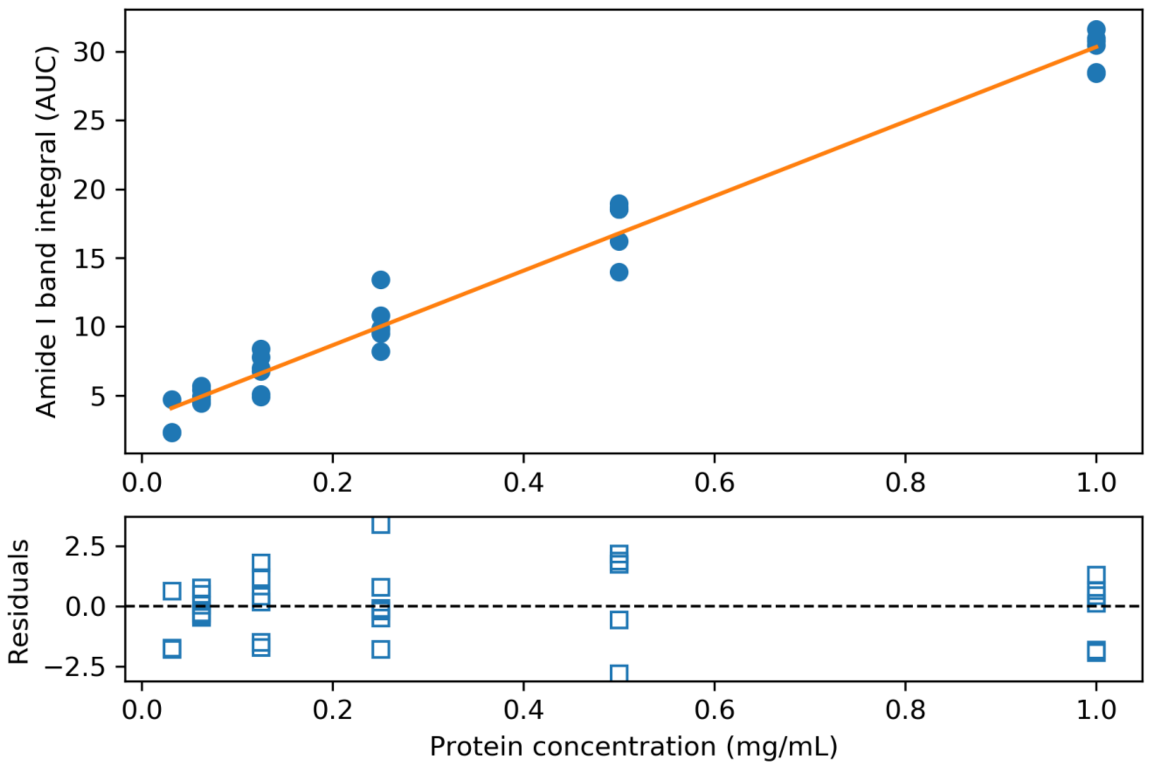

Fig. S1 Protein standard plot with the fitting residuals. The result of the F-test are listed below

Weighted residual sum of squares : SS(w)r = 36.4696
Weighted sum of squares for pure error: SS(w)e = 33.0000
Sum of squares due to lack of fit : SS(w)r - SS(w)e = 3.4696
Mean squares due to pure error : MS(w)e = 1.1000
Mean squares due to lack of fit : MS(w)lof = 0.8674
Number of points in x : n = 6
Number of independent measurements : p = 6
F statistics : MS(w)lof / MS(w)e = 0.7886
F(n-2=4, n(p-1)=30) 95% percentile : 2.6896
Fit results:
 slope: (27.1 ± 0.5) AUC/(mg/mL)
 intercept: (3.2 ± 0.2) AUC

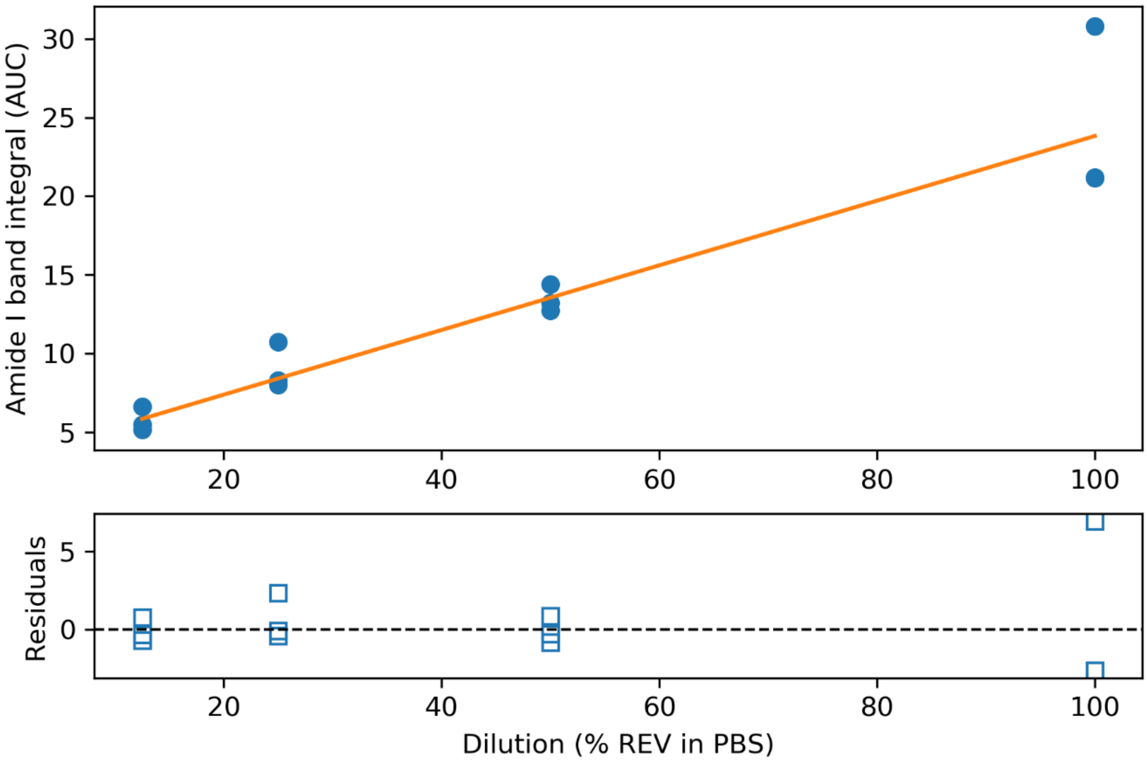

Fig. S2 Amide I AUC plot vs. red blood cell derived extracellular vesicle (REV) dilution test (presented as %REV in PBS). The result of the F-test are listed below

Weighted residual sum of squares : SS(w)r = 12.8773
Weighted sum of squares for pure error: SS(w)e = 12.0000
Sum of squares due to lack of fit : SS(w)r - SS(w)e = 0.8773
Mean squares due to pure error : MS(w)e = 1.5000
Mean squares due to lack of fit : MS(w)lof = 0.4386
Number of points in x : n = 4
Number of independent measurements : p = 3
F statistics : MS(w)lof / MS(w)e = 0.2924
F(n-2=2, n(p-1)=8) 95% percentile : 4.4590
Fit results:
slope: (0.21 ± 0.01) AUC/(% REV in PBS)
intercept: (3.3 ± 0.5) AUC

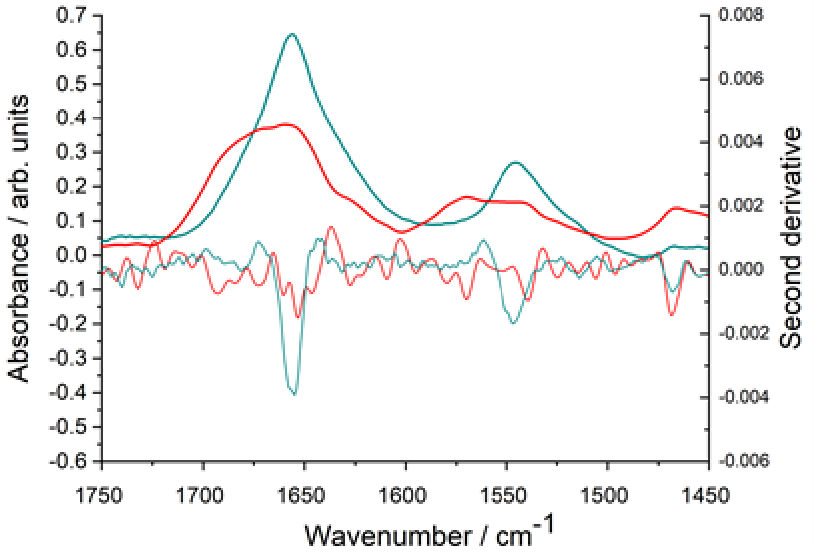

Fig. S3 Amide I and amide II spectral region of intact REV sample (green line) and after disassembling by ethanol abs. (red line). For better visualization of changes in secondary structure, the second derivative spectra are also included: while the intact REV sample contains proteins mainly with helical structure (thinner green line), after treatment with ethanol high amount of β-sheets, turns and disordered motifs of aggregated and/or denaturated proteins appear (thinner red line)
